# Identification of Novel Bioactive Compound Derived from *Rheum officinalis* against *Campylobacter jejuni* NCTC11168

**DOI:** 10.1155/2020/3591276

**Published:** 2020-07-01

**Authors:** Mohammed Yosri, Basma H. Amin, Nermine N. Abed, Amal S. Elithy, Sayed M. Kareem, Nagwa M. Sidkey

**Affiliations:** ^1^The Regional Center for Mycology and Biotechnology, Al-Azhar University, Nasr City, Cairo 11787, Egypt; ^2^Faculty of Science (Girls Branch), Al-Azhar University, Nasr City, Cairo, Egypt

## Abstract

Gastric diseases are increasing with the infection of *Campylobacter jejuni.* Late stages of infection lead to peptic ulcer and gastric carcinoma. *C. jejuni* infects people within different stages of their life, especially childhood, causing severe diarrhea; it infects around two-thirds of the world population. Due to bacterial resistance against standard antibiotic, a new strategy is needed to impede *Campylobacter* infections. Plants provide highly varied structures with antimicrobial use which are unlikely to be synthesized in laboratories. A special feature of higher plants is their ability to produce a great number of organic chemicals of high structural diversity, the so-called secondary metabolites. Twenty plants were screened to detect their antibacterial activities. Screening results showed that *Rheum officinalis* was the most efficient against *C. jejuni*. Fractionation pattern was obtained by column chromatography, while the purity test was done by thin-layer chromatography (TLC). The chemical composition of bioactive compound was characterized using GC-MS, nuclear magnetic resonance, and infrared analysis. Minimal inhibitory concentration (MIC) of the purified compound was 31.25 *µ*g/ml. Cytotoxicity assay on Vero cells was evaluated to be 497 *µ*g/ml. Furthermore, the purified bioactive compound activated human lymphocytes *in vitro*. The data presented here show that *Rheum officinalis* could potentially be used in modern applications aimed at the treatment or prevention of foodborne diseases.

## 1. Introduction

People at several stages of their life are infected by gastrointestinal bacteria including *Campylobacter jejuni* and *Helicobacter pylori* [1, 2]. *C. jejuni* is a principle factor for diarrhea in developed countries [3, 4].

*C. jejuni* primarily infects large intestine and the distal portion of the small intestine so that it is able to cause changes in the environment around it by reducing the acidity; it interacts with host mucosa by surface adhesion proteins triggering proinflammatory responses, causing intestinal metaplasia, and develops at late stages to gastric adenocarcinoma [5, 6]. It transmits to human by consuming either contaminated milk or raw meat [7, 8].

Due to bacterial resistance to antibiotics, a new alternative compound that possesses antibacterial activity should be found [9]. Medicinal plants have a large bank of rich and complex compounds which are unlikely to be synthesized in laboratories. Natural products from medicinal plants induce new agents or antimicrobial use. A special characteristic of these plants lies in their ability to produce a large number of organic chemicals of high structural diversity, which are called secondary metabolites. This study is aiming to find an alternative strategy to limit *Campylobacter jejuni* contamination and to impede *Campylobacter jejuni* infections using natural products [10, 11].

## 2. Materials and Methods

### 2.1. Bacteria and Media

*C. jejuni* NCTC 11168 was kindly obtained from the Department of Reproductive Diseases, Animal Reproduction Research Institute (ARRI), Giza, Egypt. Prior to test, this strain was cultured on brain heart infusion agar supplemented with 10% (v/v) sterile defibrinated sheep blood and incubated at 42°C for 24 h in microaerobic conditions (5% O_2_, 10% CO_2_, and 85% N_2_) in CO_2_ Incubator, Fisher, Germany.

### 2.2. Plant Collection and Extraction

Twenty plant roots were collected from traditional medicine shops as shown in Table 1. All plants were rinsed, sliced, dried (50°C for 24 h), ground, and sieved (80 mesh); the fine particles were stored in a clean container for further analysis. Pure methanol, hexane, and sterile distilled water were used as solvents for extraction. Each solvent (500 ml) was added to 50g of each powdery plant material and homogenized for 20 min using homogenizer and then allowed to stand for 1 hour. Extracts were passed through a Whatman filter paper No. 1. Mixtures were then centrifuged at 6000 rpm for 10 min to obtain clear extracts. Solvents were allowed to evaporate completely using a rotary evaporator, Fisher, USA. Then, the final extracts of each plant were completed up to 5 ml of each solvent [12–14].

### 2.3. Antibacterial Assay

In brief, about 20 ml of the medium was poured into sterile plates (9 cm) and allowed to solidify, and 5 mm diameter holes were cut in the agar using sterile cork borer (plates were in triplicate sets for each plant extract). Plates were inoculated by 0.5 ml of fixed inoculum of *Campylobacter jejuni* NCTC11168 (600 cell/ml) according to [15]. The inoculum was streaked over the surface of blood agar medium. Plates were dried for 30 min. Holes were filled by 100 *µ*l of each concentrated plant extract filtrate. Negative control wells were loaded with the specific plant extract solvent; plates were left in a cooled refrigerator at 4°C for one hour for diffusion; then the plates were incubated in the CO_2_ incubator. At the end of the incubation period, the inhibition zones were measured at three points along the diameter of the plate and the mean was calculated; the inhibition zones in control sets were compared with those of various treatments [16, 17].

### 2.4. Extraction and Solvent-to-Solvent Fractionation

Crude extract was partitioned by extraction with different solvents in order to subfractionate the plant components according to their polarity: hexane, chloroform, ethyl acetate, *n*-butanol, benzene, toluene, and methanol. Every fraction was tested for their antimicrobial activity by well diffusion assay. Controls were prepared for each fraction by drying the same amount of solvents and following the same subfractionation method without plant extract as reported in [18].

### 2.5. Separation of the Active Crude Plant Extract Using Column Chromatography

A column about 2 × 25 cm was used for this purpose. Insert a piece of cotton in the tapering lower end of column. Pack the column with 10g silica gel (mix silica gel with chloroform and pour the suspension into column in portions). Allow each part to settle before adding more suspension but without leaving the silica gel to dry. The column was left for 24 h for complete settling. Only 2 ml of the methanol crude extract was applied cautiously on the top of the column to be fractionated [19].

The column was eluted with gradient solvents using 50 ml volume of the following solvents: hexane, hexane:chloroform (1 : 1 v/v); chloroform:ethyl acetate (1 : 1 v/v); chloroform:ethyl acetate (1 : 2 v/v); ethyl acetate:methanol (3 : 1 v/v), ethyl acetate:methanol (2 : 1 v/v); ethyl acetate:methanol (1 : 1 v/v); and methanol. Fractions were collected each of 3 ml. The resultant fractions were analyzed by thin-layer chromatography to check their purity using two solvent systems consisting of toluene:ethyl acetate:formic acid (5 : 4:1 v/v) and chloroform : acetone : isopropanol (5 : 4 : 1) [20].

Then, 10 *µ*l of the resultant fractions was spotted on silica gel plates, which were developed for 2 hrs. After development of the plates, the plates were dried at room temperature. The spots were visualized under UV light. Fractions were collected where about 24 fractions were tested for their biological activity against *C. jejuni* NCTC11168.

### 2.6. Determination of the Minimum Inhibitory Concentration (MIC) for the Purified Compound

Twofold serial dilutions of the active compound were prepared and tested for their biological activity against *C. jejuni* NCTC11168 [21].

### 2.7. Evaluation of Cytotoxic Effects of the Most Active Fraction against VERO Cell Line

Fresh medium containing different concentrations of the test sample was added after 24 h of seeding. The microtiter plates were incubated at 37°C in a humidified incubator with 5% CO_2_ for a period of 24 h. Three wells were used for each concentration of the test sample. Control cells were incubated without test sample and with or without DMSO. After incubation of the cells, viable cells yield was determined by a colorimetric method using a test wavelength of 490 nm [22].

### 2.8. *In Vitro* Lymphocyte Activation Assay

To separate lymphocytes, saline solution was added to healthy blood (1 : 1 v/v) then carefully overlaid over Ficoll gradient media which then was centrifuged at 1500×*g* for 15 min. Thin-layer medium was carefully withdrawn then cultured on RPMI medium. The most bioactive fraction was purified and dissolved in dimethyl sulfoxide (DMSO) then cultured with separated cells at 37°C for 24 h [23].

### 2.9. Transmission Electron Microscopy Preparation

Cells were centrifuged at 2000×*g* for 10 min; then residual cells were fixed in 3% glutaraldehyde in 0.1 M sodium cacodylate buffer (pH 7.0) for 2 h at room temperature, rinsed in the same buffer, and postfixed in 1% osmium tetroxide for 2 h at room temperature. The samples were dehydrated in an ethanol series ranging from 10% to 90% for 15 min in each alcohol dilution and finally with absolute ethanol for 30 min. Samples were infiltrated with epoxy resin and acetone through a graded series till finally in pure resin. Ultrathin sections were collected on Formvar-coated copper grids. Sections were then double-stained in uranyl acetate followed by lead citrate. Stained sections were observed with a JEOL JEM 1010 transmission electron microscope at 70 kV at the Regional Center for Mycology and Biotechnology (RCMB), Al-Azhar University [24].

### 2.10. Determination of the Chemical Properties by Spectroscopic Analysis of the Purified Antibacterial Agent

(A) Mass spectroscopy: mass spectroscopy was carried out using Direct Inlet Unit (DI-50) of SHIMADZU GC/MS-QP5050 A. Software: Class 5000. Ionization model: EI. Ionization voltage: 70 ev. Scan speed: 2000 amu/sec. Scan interval: 0.5 sec, at the Regional Center for Mycology and Biotechnology, Al-Azhar University

(B) Infrared (IR) spectra: infrared spectrum of the new antimicrobial agent was conducted in potassium bromide using Fourier Transform infrared and Pye Unicam SP300 IR spectrophotometer at the Micro-Analytical Center, Cairo University

(C) The proton nuclear magnetic resonance (^1^H-NMR): ^1^H-NMR was conducted in deuterated chloroform using Varian Gemini 200 and 300 MHz NMR spectrophotometer at the Micro-Analytical Center, Cairo University.

## 3. Results and Discussion

The inhibitory effect of twenty plant extracts was determined using well diffusion assay and mean diameters of inhibition were recoded as shown in Table 1. Screening revealed that plants vary in their activity against test microbe. It was found that *Rheum officinalis* had the most active anti-*Campylobacter* agent. It was recognized centuries ago that *Rheum* contained active components like anthraquinones, stilbenes, dianthrones, anthocyanins, flavonoids, galloyl-glucoses, organic acids, phenylbutanones, etc., thus having pharmacological activity [25].

Data revealed that plant extracts had different activities against test microbe where *Rosmarinus officinalis, Artemisia*, *Commiphora myrrha*, *Cassia angustifolia*, and *Rheum officinalis* exhibited highest activities, while plant extract *Rheum officinalis* contained the most effective anti-*Campylobacter* agent as shown in Table 1.

Extraction with different solvents indicated that methanol was the solvent giving highest growth inhibition action. So, it was used in chromatographic assays as shown in Table 2. In view of the findings of other researchers, various solvents were used for extracting the biosynthesized antimicrobial agents, versus methanol [26], chloroform [27], and ethyl acetate [18, 28].

**Figure 1:**
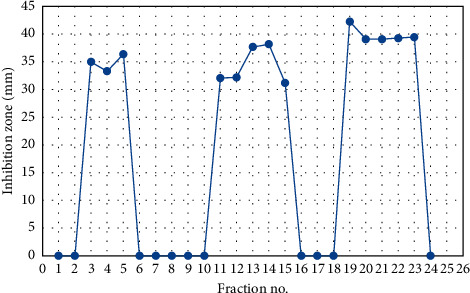
Fractionation pattern of antibacterial agents produced by *Rheum officinalis*, using column chromatography, and their effect against *C. jejuni* NCTC11168.

Chromatographic assays indicated that fractions vary in their biological activity against tested microbes where fractions 3–5, fractions 11–15, and fractions 19–23 showed promising antibacterial activities, while fraction 19 was the most active compound against *C. jejuni* NCTC11168 as shown in Table 3 and Figure 1. In view of the findings of other scientists, column chromatography was packed with silica gel and an eluting solvent composed of various ratios of the solvent system was used for fractionation of the crude extract into active fractions [18]. Separated compounds differ in their activity against test organism. Purification of separated compounds on TLC plates using different solvent systems was utilized, while polar solvents achieved higher antimicrobial activity compared to nonpolar solvents in accordance with [29].

In the present investigation, minimal inhibitory concentration of the antimicrobial agent produced from *Rheum officinalis* against *C. jejuni* NCTC11168 was 31.25 *µ*g/ml as shown in Table 4. This result was in complete accordance with [30] for evaluating the antibacterial activity of Thai medicinal plants.

Cytotoxicity assay indicated that *Rheum officinalis* has no cytotoxic effects on Vero cells as proposed by [31] where CC_50_ of *Rheum officinalis* was 497 *µ*g/ml. *Rheum officinalis* had a very effective antibacterial fraction and induced immune responses by the activation of lymphocytes; these results were in accordance with [32].

**Figure 2:**
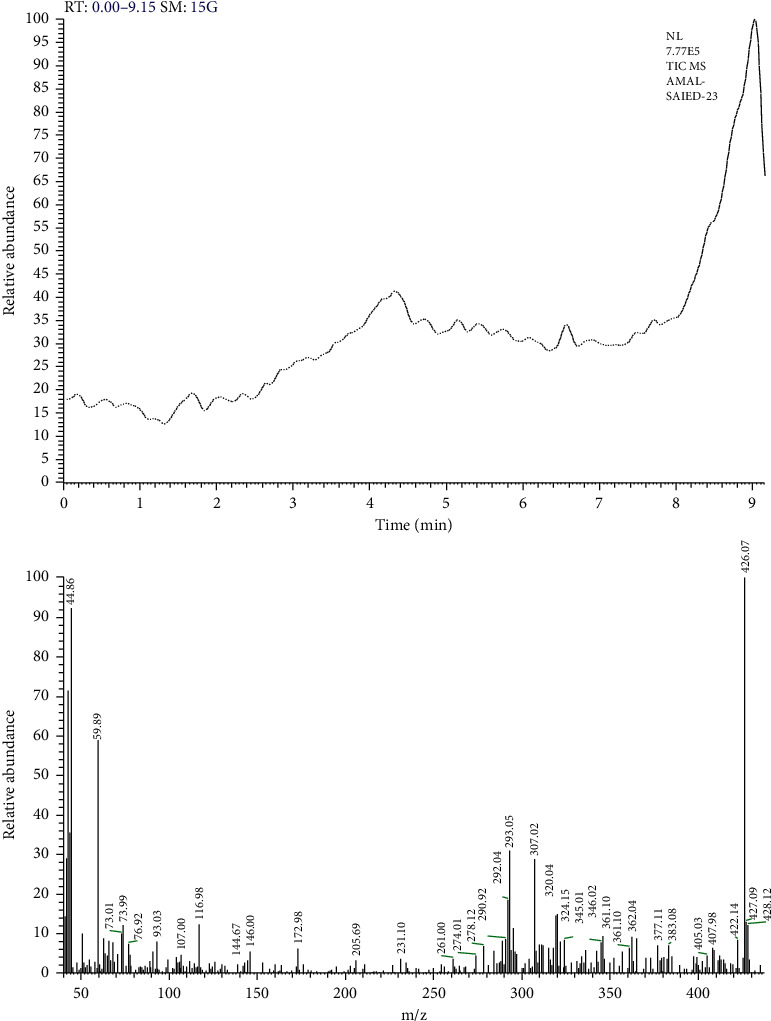
GC-MS of the purified bioactive compound from *Rheum officinalis* against *C. jejuni* NCTC11168.

The purity of the antibacterial compound was checked by chromatographic separation on silica gel TLC plate that showed one band under short wavelength. Furthermore, the purity of this compound was confirmed by the total ion chromatogram resulting from mass spectroscopic (MS) analysis of this substance that was separated in a single peak for pure compound as shown in Figure 2, and the mass spectrum showed molecular ion peak at m/z 44.86 (92.39%), 59.89 (58.96%), 73.01 (10.02%), 73.99 (12.23%), 76.92 (7.36%), 93.03 (8.03%), 107.00 (4.61%), 116.98 (12.48%), 144.67 (3.18%), 146.00 (5.55%), 172.98 (6.16%), 205.6 (3.22%), 231.10 (3.68%), 261.00 (3.71%), 274.01 (4.41%), 278.12 (6.86%), 290.92 (8.62%), 292.04 (18.46%), 293.05 (30.96%), 307.02 (28.96%), 320.04 (14.89%), 324.15 (8.34%), 345.01 (7.88%), 346.02 (9.38%), 361.10 (6.51%), 362.04 (9.21%), 377.11 (7.09%), 383.08 (7.07%), 405.03 (4.50%), 407.98 (6.53%), 422.14 (8.36%), 427.09 (13.01%), 428.12 (12.21%), and base peak at m/z 426.07 (100.00%).

**Figure 3:**
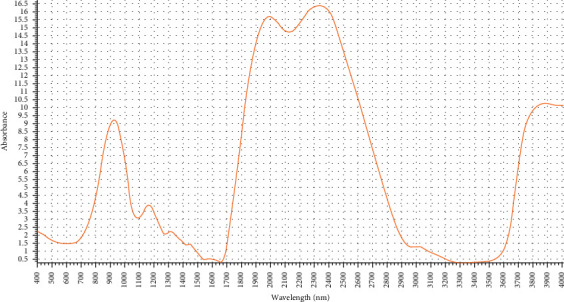
IR spectrum of the purified bioactive compound from *Rheum officinalis* against *C. jejuni* NCTC11168.

Infrared (IR) spectrum of this compound had absorption bands at 2945 due to the presence of C-H aliphatic; band at 1595 due to the presence of C=N bonding; band at 1650 due to the presence of C=O group of ester; band at 1530 due to the presence of C=C bonding; band at 1280 due to the presence of C-N bonding; and band at 1080 due to the presence of C-H aromatic as shown in Figure 3.

**Figure 4:**
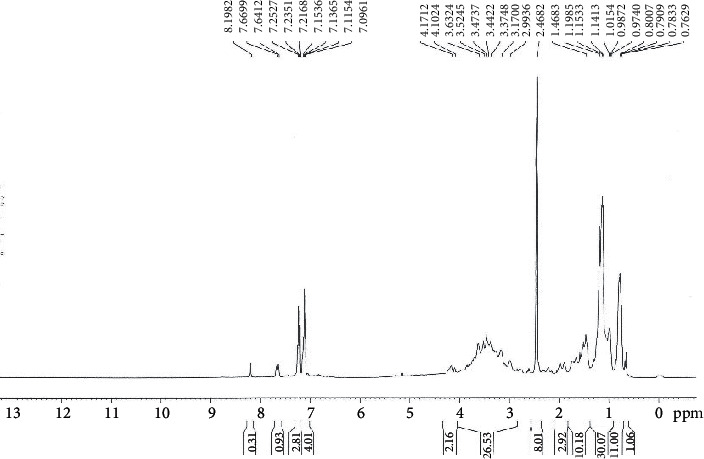
NMR spectrum of the purified bioactive compound from *Rheum officinalis* against *C. jejuni* NCTC11168.

The nuclear magnetic resonance (NMR) spectrum of the compound that dissolved in DMSO-d6 is illustrated in Figure 4; it showed signals at 7.09 and 7.66 ppm indicating the presence of aromatic-H; signals at 0.79 ppm indicate the presence of CH_2_-CH_3_ bonding; signals at 1.15 ppm indicate the presence of CH_3_ group; and signals at 1.46 ppm indicate the presence of aromatic-CH_2_.

**Figure 5:**
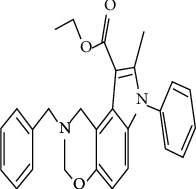
Expected formula of the purified compound from *Rheum officinalis* tested against *C. jejuni* NCTC11168 (8-benzyl-2-methyl-3-phenyl-3,7,8,9-tetrahydro-6-oxa-3,8-diaza-cyclopenta[A]naphthalene-1-carboxylic acid ethyl ester).

Consequently, the expected molecular formula for this compound is C_27_ H_26_ N_2_ O_3_ and the suggested chemical name is 8-benzyl-2-methyl-3-phenyl-3,7,8,9-tetrahydrooxa3,8diazacyclopenta[A]naphthalene-1-carboxylic acid ethyl ester. The proposed chemical structure is illustrated in Figure 5.

In view of the findings of other investigators, *Rheum officinalis* is a medicinal herb with clinical practice act as antipyretic, antibacterial, hemostatic, and antineoplastic; this is due to its bioactive components like flavonoids, organic acids, phenyl-butanones, galloyl-glucoses, stilbenes, and anthocyanin [33]. However, [34] reported that *Rheum officinalis* possessed pharmacological activities due to its phytochemical constituents, thus acting as antioxidants by scavenging free radicals, anticancer effects via inhibiting the cellular proliferation, anti-inflammatory activities through attenuating the activity of TNF-*α*, NF-*к*B, IL-2, and IL-6, and antidiabetic activity via decreasing the activity of glucose-6-phosphatase, fructose-1,6-diphosphatase, and aldolase. On the other hand, raspberry ketone (RK) from *Rheum officinalis* inhibited melanogenesis by regulation of the posttranscriptional of tyrosinase gene expression [35]. In addition, *Rheum officinalis* contained tannins and gallic acid which act as antioxidant phenolic components [36].

Moreover, the authors of [37] reported that *Rheum* spp. contained flavan (3′,5′,5,7-tetrahydroxyflavanone), pyrones (progallin A), anthrones (10S-3-methyl-1,8,10-trihydroxy-10-*β*-D-glucopyranosyl-9(10H)-anthracene), and acyl glycosides (4-(4′-hydroxyphenyl)-2-butanone-4′-O-*β*-D-glucopyranoside) which are used for treating accumulation and purging, draining damp heat, and cooling blood. Also, [38] indicated that polyphenol contents from *Rheum officinalis* were effective against Gram-positive bacteria (*Staphylococcus* spp.). Furthermore, [39] reported that methyl gallate and its derivatives extracted from *Acacia farnesiana* showed activity against *C. jejuni* with MIC 50 *μ*g/ml. However, our report identified a compound with lower MIC which suggests better antibacterial activity. Moreover, it is recommended to have different bioactive compounds from different sources to be suitable for a wide range of patients.

Additionally, [40] indicated that *Rheum* spp. has antimicrobial activity against a wide range of pathogens including *Candida albicans* DSMZ 1386, *Enterococcus durans*, *Enterococcus faecalis* ATCC 29212, *Escherichia coli* ATCC 25922, *Klebsiella pneumoniae*, *Listeria monocytogenes* ATCC 7644, *Pseudomonas fluorescence* P1, *Salmonella enteritidis* ATCC 13075, *Salmonella infantis*, *Salmonella typhimurium* SL 1344, and *Staphylococcus epidermidis* DSMZ 20044.

**Figure 6:**
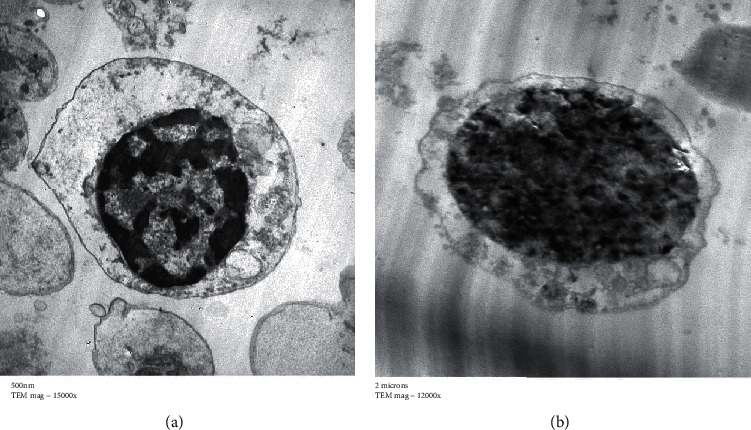
Effect of the purified compound from *Rheum officinalis* on isolated human lymphocytes. (a) Control. (b) Treated.

In the current study, purified compound activated isolated human lymphocytes as shown in Figure 6. Also, the authors of [41] reported that *Rheum officinalis* has an immense potential in healthcare as it is regulating gastrointestinal tract, protecting cardiovascular system, and having anticancer, antimicrobial, anti-inflammatory activities, and hepatoprotective activities.

This is the first documentary of the produced compound of 8-benzyl-2-methyl-3-phenyl-3,7,8,9-tetrahydro-6-oxa-3,8-diaza-cyclopenta[A]naphthalene-1-carboxylic acid ethyl ester produced by *R. officinalis* in the literature.

## 4. Conclusion

In the current study, chemical composition of bioactive compound derived from of *Rheum officinalis* against *Campylobacter jejuni* was identified and elucidated using different solvent systems to isolate the most bioactive compound. Therefore, this approach is successful for the standardization of methods and establishment of results reported for pharmaceutical activities of *Rheum officinalis*. Further investigations are recommended to test other promising fractions for other future applications.

## Figures and Tables

**Table 1:** Screening of the anti-*Campylobacter jejuni* activity of different plant extracts.

Collected plants	Inhibited zone H_2_O extract	Inhibited zone methanol extract	Inhibited zone hexane extract
*Salvia officinalis*	8.03 ± 0.25	15.1 ± 0.00	12.0 ± 0.00
*Rosmarinus officinalis*	26.8 ± 0.51	18.0 ± 0.00	14.7 ± 0.00
*Foeniculum vulgare*	11.2 ± 0.00	20.1 ± 0.00	11.5 ± 0.00
*Hordeum vulgare*	10.3 ± 0.00	15.1 ± 0.00	20.6 ± 0.00
*Artemisia*	31.7 ± 0.00	20.4 ± 0.00	18.6 ± 0.00
*Anethum graveolens*	12.5 ± 0.00	15.2 ± 0.00	12.2 ± 0.00
*Costus*	7.7 ± 0.00	15.2 ± 0.00	17.3 ± 0.00
*Nigella sativa*	8.5 ± 0.00	10.6 ± 0.00	15.3 ± 0.00
*Commiphora myrrha*	16.4 ± 0.00	19.7 ± 0.00	30.2 ± 0.00
*Thymus vulgaris*	9.5 ± 0.00	9.3 ± 0.32	9.2 ± 0.00
*Matricaria chamomilla*	8.4 ± 0.00	15.1 ± 0.00	8.0 ± 0.00
*Achillea*	23.2 ± 0.00	8.2 ± 0.30	8.4 ± 0.00
*Plantago uniflora*	10.2 ± 0.32	10.5 ± 0.00	11.4 ± 0.20
*Cassia angustifolia*	28.2 ± 0.30	15.7 ± 0.00	23.1 ± 0.20
*Rheum officinalis*	24.1 ± 0.00	39.1 ± 0.00	20.2 ± 0.00
*Cinnamomum zeylanicum*	10.5 ± 0.00	15.4 ± 0.00	15.4 ± 0.00
*Allium sativum*	10.4 ± 0.00	10.1 ± 0.00	13.2 ± 0.00
*Lepidium sativum*	20.0 ± 0.2	9.9 ± 0.00	9.5 ± 0.35
*Trigonella foenum-graecum*	16.8 ± 0.00	19.2 ± 0.1	30.4 ± 0.00
*Senegalia senegal*	17.5 ± 0.15	22.4 ± 0.00	23.4 ± 0.00

**Table 2:** Comparative antibacterial activity of different solvents extraction to *Rheum officinalis*.

Solvent	Inhibition zone of crude (mm)	Inhibition zone of solvent (mm)
Hexane	20.2 ± 0.0	0.0
Chloroform	12.3 ± 0.1	0.0
Ethyl acetate	15.1 ± 0.14	7.0 ± 0.0
*n*-Butanol	15.0 ± 0.0	0.0
Benzene	13.0 ± 0.0	0.0
Toluene	18.0 ± 0.0	0.0
Methanol	39.1 ± 0.0	0.0
Fats	9.0 ± 0.0	0.0

**Table 3:** Fractionation pattern of antibacterial agents produced by *Rheum officinalis*, using column chromatography, and their effect against *C. jejuni* NCTC11168.

Fraction no.	Inhibited zone (mm)
1	0.00
2	0.00
3	35 ± 0.00
4	33.3 ± 0.05
5	36.4 ± 0.11
6	0.00
7	0.00
8	0.00
9	0.00
10	0.00
11	32.1 ± 0.00
12	32.2 ± 0.00
13	37.7 ± 0.00
14	38.2 ± 0.00
15	31.2 ± 0.00
16	0.00
17	0.00
18	0.00
19	42.3 ± 0.1
20	39.1 ± 0.00
21	39.1 ± 0.00
22	39.3 ± 0.00
23	39.46 ± 0.15
24	0.00

**Table 4:** Screening of minimal inhibitory concentration of different concentrations of *Rheum officinalis* against *C. jejuni* NCTC11168.

Concentration (*µ*g/ml)	Inhibited zone (mm)
500	42.5 ± 0.0
250	37.5 ± 0.0
125	28.6 ± 0.0
62.50	17.5 ± 0.0
31.25	11.0 ± 0.0
15.62	0
7.81	0
3.90	0
1.95	0
0.97	0

## Data Availability

All the raw data included in the manuscript are available from the corresponding author upon reasonable request.
